# Effect of ECOG performance status on outcomes in patients with acute myeloid leukemia and other high-grade myeloid neoplasms

**DOI:** 10.1038/s41375-022-01745-4

**Published:** 2022-11-25

**Authors:** Gabrielle Paras, Megan Othus, Kelda Schonhoff, Carole Shaw, Mohamed Sorror, Anna B. Halpern, Jacob Appelbaum, Paul Hendrie, Roland B. Walter, Elihu H. Estey, Mary-Elizabeth M. Percival

**Affiliations:** 1 Department of Medicine, University of Washington, Seattle, WA USA; 2 Public Health Sciences Division, Fred Hutchinson Cancer Center, Seattle, WA USA; 3 Clinical Research Division, Fred Hutchinson Cancer Center, Seattle, WA USA; 4 Department of Laboratory Medicine & Pathology, University of Washington, Seattle, WA USA; 5 Department of Epidemiology, University of Washington, Seattle, WA USA

**Keywords:** Acute myeloid leukaemia, Disease-free survival

## Introduction

For acute myeloid leukemia (AML), survival remains poor among older patients due to significant comorbidities and poorer performance status (PS) [1]. This finding likely reflects an association with genomic abnormalities predictive of resistance to current therapies and a propensity for death within 28 days of treatment (treatment-related mortality or TRM) [2, 3]. Poor PS is an important determinant of TRM. The widely-used Eastern Cooperative Oncology Group (ECOG) system assigns bed-ridden patients PS 4, patients in bed >50% of the usual waking hours PS 3, and patients who are able to perform the great majority of their usual activities PS 0 or 1, with the latter somewhat more impaired than the former [4].

Clinical trials typically restrict participation to patients with PS 0–2, assuming the risks of TRM and other severe toxicities are likely unacceptable in patients with PS 3–4 or simply to enroll lower risk patients. Patients with PS 0 and PS 1 comprise the majority of those treated in oncology clinical trials. Here we examine whether response and survival outcomes vary between patients with low ECOG PS who are generally combined for trial eligibility and study analysis.

## Methods

### Patient population

Our database identified 1252 patients with newly diagnosed WHO-defined AML or high-grade myeloid neoplasms (≥10% blasts, whom we typically treat as AML) who qualified for disease-directed therapy at the University of Washington (UW)/Fred Hutchinson Cancer Center between January 1, 2008 and May 31, 2018. The study was approved by the UW Institutional Review Board. Also available were pre-treatment data on age, sex, PS, de novo vs secondary AML, ELN 2017 cytogenetic risk [5], TRM score [2], induction treatment intensity, hematopoietic cell transplantation-specific comorbidity index (HCT-CI; available in a subset of 597 patients) [6], trial status, and baseline values for albumin, white blood cells (WBC), platelets, and creatinine. Only patients listed as ECOG PS 0–2 in the database were included; no upper age cutoff was used. Patients were considered to have secondary disease if they had previously received chemotherapy or had an antecedent hematologic disorder.

Survival at 4 and 8 weeks after beginning treatment was measured. Complete remission (CR) after induction chemotherapy and CR with incomplete hematologic recovery (CRi) were defined using 2017 ELN criteria [5]. Measurable residual disease (MRD) was assessed using multiparameter flow cytometry [7]. Induction treatment was classified into 2 levels of intensity: low [“hypomethylating” agents (HMA)] and high (such as 7 + 3, but most commonly FLAG or G-CLAM) [8].

### Statistical analysis

Overall survival (OS) was measured from the first day of induction therapy. Fisher’s exact test and Wilcoxon rank sum tests were used to evaluate associations between PS and other categorical and quantitative covariates, respectively. Univariate and multivariable linear regression models were performed to evaluate covariate associations and 4- and 8-week survival. Data were analyzed using R version 4.1.1 (R Development Core Team, Vienna, Austria).

## Results

### Patient characteristics

Among the 1252 patients included, 997 (79.6%) had PS 1; 46 patients (3.7%) had PS 0, and 209 patients (16.7%) had PS 2 (Table 1). Median age increased with increasing PS category (58 versus 62 versus 64 years); median age of the entire population was 65 years. Other significant associations with increasing PS included HCT-CI score of 5 + , higher TRM score, increased median WBC and creatinine, whereas median albumin decreased with increasing PS category. Patients with lower PS scores were more likely to receive high intensity therapies (*p* < 0.001). Regardless of PS, the majority of patients were treated off clinical trials, but there was a higher percentage of PS 0 patients on trials (33%) compared to PS 1 (19%) and PS 2 (9%) (*p* < 0.001).

**Table 1:** Univariate associations between patient characteristics and PS. Median (range) or *N* (%) reported for summary.

Factor	PS 0 (*n* = 46)	PS 1 (*n* = 997)	PS 2 (*n* = 209)	*P*-value
Age (years)	58 (21, 82)	62 (19, 92)	64.2 (19, 92)	0.027
Age ≤75	40 (87)	833 (84)	162 (78)	0.093
Age >75	6 (13)	164 (16)	47 (22)	
Gender				
Female	19 (41)	415 (42)	90 (43)	0.93
Male	27 (59)	582 (58)	119 (57)	
Blasts				
10–20% blasts	11 (24)	160 (16)	24 (11)	0.0045
20% or more blasts	27 (59)	583 (58)	109 (52)	
Missing data	8 (17)	254 (25)	76 (36)	
Disease status				
De novo	17 (37)	416 (42)	91 (44)	0.74
Secondary	29 (63)	581 (58)	118 (56)	
Treated on or off trial				
Off trial	31 (67)	807 (81)	190 (91)	<0.001
On trial	15 (33)	190 (19)	19 (9)	
ELN 2017 risk				
Intermediate	20 (43)	312 (32)	40 (20)	<0.001
Favorable	6 (13)	149 (15)	30 (15)	
Adverse	12 (26)	272 (28)	58 (28)	
Missing data	8 (17)	254 (26)	76 (37)	
TRM score total	1.7 (0, 6.2)	5.8 (0, 77.7)	15.7 (0.1, 90)	<0.001
TRM categories				
TRM 0–3.9	42 (91)	420 (42)	23 (11)	<0.001
TRM 4–6.9	4 (9)	272 (27)	26 (13)	
TRM 7–100	0 (0)	300 (30)	158 (76)	
Albumin	4 (2.8, 4.9)	3.7 (1.3, 5.2)	3.4 (1.8, 4.7)	<0.001
WBC	17.3 (0.3, 252.8)	22.2 (0.2, 493.7)	26.1 (0.3, 341.2)	0.015
Platelets	121 (4, 906)	89.8 (1, 1792)	80.1 (2, 727)	0.055
Creatinine	0.9 (0.5, 1.7)	1 (0.4, 11.6)	1.2 (0.3, 8.3)	0.0061
Therapy intensity				
High	32 (70)	654 (66)	118 (56)	<0.001
Low	13 (28)	201 (20)	40 (19)	
No treatment/palliative	0 (0)	66 (7)	28 (13)	
Unknown/no treatment here	1 (2)	76 (8)	23 (11)	
HCT-CI total	3 (0, 9)	3.8 (0, 13)	5.8 (1, 13)	<0.001
HCT-CI 0	2 (8)	32 (7)	0 (0)	<0.001
HCT-CI 1–2	9 (36)	135 (28)	13 (15)	
HCT-CI 3–4	11 (44)	150 (31)	18 (20)	
HCT-CI 5+	3 (12)	167 (35)	57 (65)	
4-week mortality				
Death within 4 weeks	0 (0)	50 (5)	20 (10)	0.012
8-week mortality				
Death within 8 weeks	0 (0)	99 (10)	44 (22)	<0.001
Remission				
CR without MRD	23 (52)	383 (46)	57 (38)	0.12
CR with MRD, CRi, or resistant	21 (48)	449 (54)	92 (62)	
Remission				
CR + CRi (with or without MRD)	31 (70)	569 (68)	92 (62)	0.25
Resistant	13 (30)	263 (32)	57 (38)	

*PS* performance status, *ELN* European leukemianet, *TRM* treatment-related mortality, *WBC* white blood cells, *HCT-CI* hematopoietic cell transplantation-specific comorbidity index, *CR* complete remission, *CRi* complete remission with incomplete hematologic recovery, *MRD* measurable residual disease.

### Associations between performance status and survival

Overall, 17% (213) of all patients died within 4 (70 patients) and 8 weeks (143 patients) of initiating anti-AML therapy. The 4-week unadjusted mortality rates were 0% for PS 0, 5% for PS 1, and 10% for PS 2 (*p* = 0.012). A similar trend appeared for 8-week mortality from PS 0 (0%) to PS 1 (10%) to PS 2 (22%) (*p* < 0.001, Table 1). Compared to PS 1, PS 2 was associated with significantly increased 4-week mortality (odds ratio [OR] 2.01, 95% confidence interval [CI] 1.17–3.46) and increased 8-week mortality (OR 2.47, 95% CI 1.67–3.67) in univariate analysis.

However, in multivariable analysis controlling for age, ELN risk, TRM score, and clinical trial status, when compared to PS 1, PS 2 was not associated with significantly higher risk of 4 and 8-week mortality (4-week OR 1.09, 95% CI 0.56–2.14; 8-week OR 1.46, 95% CI 0.89–2.39). Similarly, when compared to PS 0–1 combined, PS 2 was not associated with increased 4-week mortality (OR 1.67, 95% CI 0.94–2.97), though it was associated with significantly higher 8-week mortality (OR 2.26, 95% CI 1.49–3.43).

**Fig. 1:**
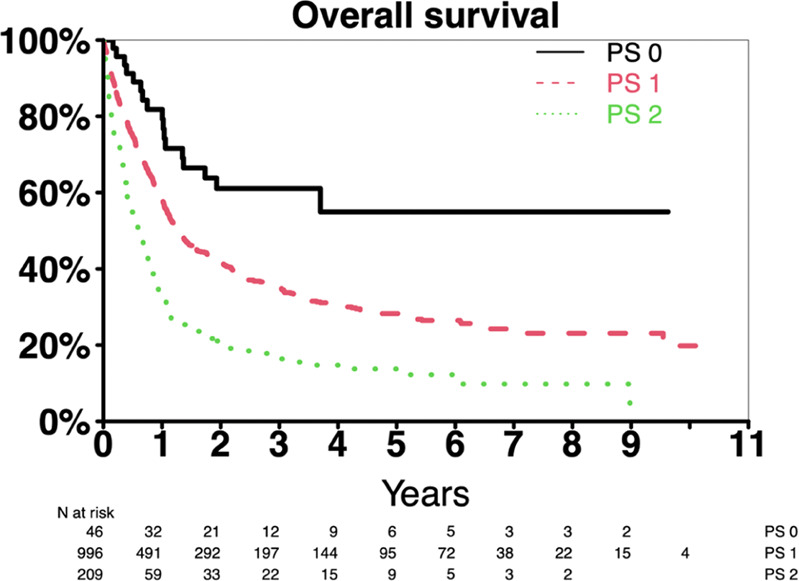
Kaplan-Meier curve for overall survival in our cohort. Survival curves for patients were stratified by performance status (PS).

In contrast to the early mortality findings, lower PS was significantly associated with increased 3-year OS with PS 0 = 61% (95% CI 48–78%), PS 1 = 35% (95% CI 32–39%), and PS 2 = 17% (95% CI 12–24%); Fig. 1.

### Relationship between performance status and remission

PS was not associated with achievement of CR (morphologic or CR without MRD), after controlling for age, secondary disease, ELN risk, TRM, clinical trial status and intensity of induction. There were no significant differences in rates of CR/CRi (with or without MRD) between PS 0 vs PS 1, PS 0 vs PS 2, or PS 0–1 combined vs PS 2.

In multivariable models, favorable cytogenetic risk (OR 3.43, 95% CI 2.15–5.46) and on trial status (OR 1.64, 95% CI 1.14–2.34) were significantly associated with increased likelihood of remission, whereas lower treatment intensity (OR 0.35, 95% CI 0.23–0.53) and secondary disease (OR 0.67, 95% CI 0.48–0.93) were associated with decreased rates of remission, consistent with the notion that the principal reason for failure to achieve CR is resistance to therapy.

## Discussion

Because most clinical trials for AML are conducted in patients with PS 0–2 [9], we attempted to assess differences in survival and achievement of CR according to specific PS score. Previous analyses found that mortality rates decline sharply 4 weeks after the start of induction therapy, suggesting that patients who die early comprise a distinctive group [2]. In our cohort, early death was a rare event, even among patients treated with high intensity therapy.

Although PS 2 was associated with significantly higher 4 and 8-week mortality in univariate analysis, the differences in mortality rates between PS 2 and PS 1 were no longer present in multivariable analysis. No significant difference was seen in 4-week mortality between PS 0–1 vs PS 2, but 8-week mortality more than doubled for PS 2, suggesting that patients with PS 1 or PS 2 can be viewed more similarly when evaluating early mortality risks. Notably, no PS 0 patients died within the first 8 weeks of treatment.

Increased PS was significantly associated with decreased 3-year OS. A similar trend was reported in a study by Ostgard et al showing that higher PS was associated with increased mortality at 3 years for PS 1 or greater compared to PS 0 [10]. Survival at 3 years is heavily influenced by achievement of an initial CR; in our analysis, we could not identify an effect of increasing PS on rate of remission after accounting for age, ELN risk, clinical trial status and treatment intensity.

One plausible reason for the similar rates of CR across PS 0–2 in our study is the number of patients with PS 0; we identified only 46 such patients (3.7% of the total cohort). Other prospective clinical trials identified a larger portion of PS 0 patients in their cohort ranging from approximately 20–40% [2, 11–14]. In contrast to our findings, evaluation of the Danish registry data found that higher PS was significantly associated with reduced rates of CR [10]. Unlike our study, the Danish study identified 26% of their population with PS 0. As evidenced by the large difference in number of patients with PS 0 between our institution and those at others, the practice of assigning ECOG PS can vary greatly amongst individual reviewers and institutions. Unless patients are asymptomatic and the diagnosis of AML was incidental, patients entered into our database are considered at least PS 1 as they usually present with symptoms when being evaluated for AML.

A possible explanation for the discrepancies in the association between higher PS and rates of remission among the two studies was the registry-based nature of the Danish study. The single center referral-based practice captured in our registry likely introduced various selection biases as well as subjectivity and local trends in assigning performance status. This subjectivity can be quantified by noting that the c-statistic value for even PS 0–2 vs PS 3–4 as a single variable was for the most part <0.6, indicating prognostic ability almost intermediate between a coin flip (c-statistic = 0.5) and perfect prediction (c-statistic = 1.0) [15]. While PS is a readily available clinical tool for assessing patient fitness for chemotherapy, incorporation of other metrics such as functional status and co-morbidities would likely be useful to improve risk stratification and assignment of treatment intensity.
